# Gene-specific DNA methylation responses to Pb^2+^ exposure in aged male zebrafish and associations with locomotor dysfunction

**DOI:** 10.1093/eep/dvag023

**Published:** 2026-06-23

**Authors:** Chia-Chen Wu, Danielle N Meyer, Grace A Winny, Dayita Banerjee, Tracie R Baker

**Affiliations:** Institute of Environmental Engineering, National Yang Ming Chiao Tung University, Hsinchu City 300093, Taiwan; Department of Environmental and Global Health, University of Florida, Gainesville, FL 32610, United States; Department of Environmental and Global Health, University of Florida, Gainesville, FL 32610, United States; Department of Environmental and Global Health, University of Florida, Gainesville, FL 32610, United States; Department of Environmental and Global Health, University of Florida, Gainesville, FL 32610, United States; University of Florida Genetics Institute, University of Florida, Gainesville, FL 32610, United States

**Keywords:** methylation, zebrafish, lead (pb2+), brain, adult

## Abstract

Lead (Pb^2+^) is a well-established neurotoxin that impairs motor, learning, and memory functions, particularly in children and younger adults. However, its impact on older adults remains less understood. Pb^2+^ toxicity involves disruption of DNA methyltransferase activity and associated epigenetic pathways, potentially altering the expression of specific genes relevant to neurological functions. As methylation patterns naturally shift during aging, Pb^2+^ exposure may induce additional neurological risks in aged populations. Using a zebrafish model, we investigated the combined effects of Pb^2+^ exposure and brain aging. Two-year-old male zebrafish were exposed to 1, 10, 100, 1000, 10 000 µg/l Pb^2+^ or fish water control for five days. Brain tissues were collected for DNA extraction and whole-genome bisulfite sequencing to assess global and gene-specific methylation changes. Our results found that Pb^2+^ exposures ≥ 100 μg/l significantly increased global methylation levels in the aged brain. Differentially methylated genes (DMGs) exhibited methylation changes within gene body regions and were mostly annotated with ion transportation and signal transduction pathways. Although only a limited number of DMGs showed corresponding changes in gene expression, several of them were associated with locomotor-related functions, including *shank1* at 10 000 Pb^2+^ μg/l, and *ptprsa, plxna2*, and *aopep* at 100 μg/l Pb^2+^. These findings suggest that Pb^2+^ exposure during aging predominantly induces gene body-localized DNA methylation changes, and the role of such epigenetic regulation in Pb-associated neurobehavioral outcomes warrants further investigation.

## Introduction

Despite decades of efforts to manage environmental exposures, lead (Pb^2+^) remains one of 10 toxicants designated by the World Health Organization (WHO) as posing major public health concerns [1]. Emerging evidence indicates that beyond its well-established neurotoxicity, Pb^2+^ can induce widespread epigenetic alterations, particularly changes in DNA methylation patterns [2–8]. DNA methylation primarily refers to the covalent addition of a methyl group to the 5th carbon of cytosine residues in DNA [9]. This epigenetic modification plays a crucial role in regulating gene expression, maintaining cellular identity, and mediating cellular responses to environmental stimuli [9], including contaminants such as Pb^2+^.

Pb^2+^ exposure has been shown to induce abnormal DNA methylation through multiple pathways, as demonstrated in the rat hippocampus [2], mouse brain [3], and human lung and breast cancer cells [4]. In addition, human embryonic stem cells exposed to Pb^2+^ exhibited methylation changes in genes involved in neurogenic signaling [5]. Pb-associated methylation changes have also been reported in epidemiological studies, including alterations in Alzheimer’s disease-related genes following occupational exposure [6], CpG site-specific methylation changes in offspring following prenatal exposure [7], and hypomethylation of interspersed DNA repeats such as Long Interspersed Nucelotide Element 1 (LINE-1) in children [10]. Furthermore, the Normative Aging Study cohort identified specific blood DNA methylation CpG sites corresponding with cumulative Pb^2+^ concentrations within bone, demonstrating a longitudinal relationship with Pb accumulation throughout the aging trajectory [8].

The exposure window of Pb^2+^ plays a critical role in shaping its health effects and associated epigenetic consequences. During early life, when neuronal cells are highly proliferative, Pb^2+^ exposure can reduce the expression of DNA methyltransferases and other regulators of methylation in the hippocampus [2]. This impairment of the methylation machinery following developmental Pb^2+^ exposure induces long-lasting DNA methylation modifications that persist into old age, leading to altered expression of neuronal maintenance genes and rendering the aging brain more susceptible to age-related degeneration [11]. Other than the effects of developmental exposure, aging itself is associated with dynamic changes in the brain’s DNA methylation landscape. In the adult brain, neurons continue to express significant levels of DNA methyltransferases, suggesting an ongoing role for DNA methylation in brain function beyond development [9]. Age-related changes in DNA methylation exhibit dynamic patterns, including neuronal activity-dependent demethylation [12]. In aged brain tissues, however, hypermethylation of synaptic genes associated with cognitive decline has been observed [13]. Nevertheless, how Pb^2+^ exposure impacts the epigenetic regulation of aged brain tissues remains poorly understood. Given the growing proportion of older adults globally [14], investigating how environmental Pb^2+^ exposures interact with the aging epigenome is critical for advancing mechanistic insight and informing public health strategies.

In this study, we hypothesized that Pb^2+^ exposure induces transcriptomic changes through methylation regulation. Our previous work demonstrated that acute Pb^2+^ exposure altered locomotor, anxiety-level behaviors, and associated transcriptomic profiles in zebrafish at 2 years of age [15]. In addition, several studies have suggested that Pb^2+^-induced behavioral impairments are more pronounced in males [16, 17]. At advanced life stages, zebrafish exhibit decreased global genome methylation compared to younger individuals [18], reflecting aging-associated epigenetic alterations. Zebrafish and humans also share common functional characteristics related to cognitive decline [19] and genome instability [18], supporting the use of zebrafish as a representative model for studying aging-related processes in human populations. Building on these findings, we conducted whole-genome bisulfite sequencing (WGBS) on the brains of 2-year-old zebrafish following 5 days of Pb^2+^ exposure, during which behavioral and transcriptomic changes were observed. Thus, this study sought to characterize Pb^2+^-induced DNA methylation alterations in the aged male zebrafish brain and examine their associations with previously observed behavioral and transcriptomic responses.

## Results

**Figure 1:**
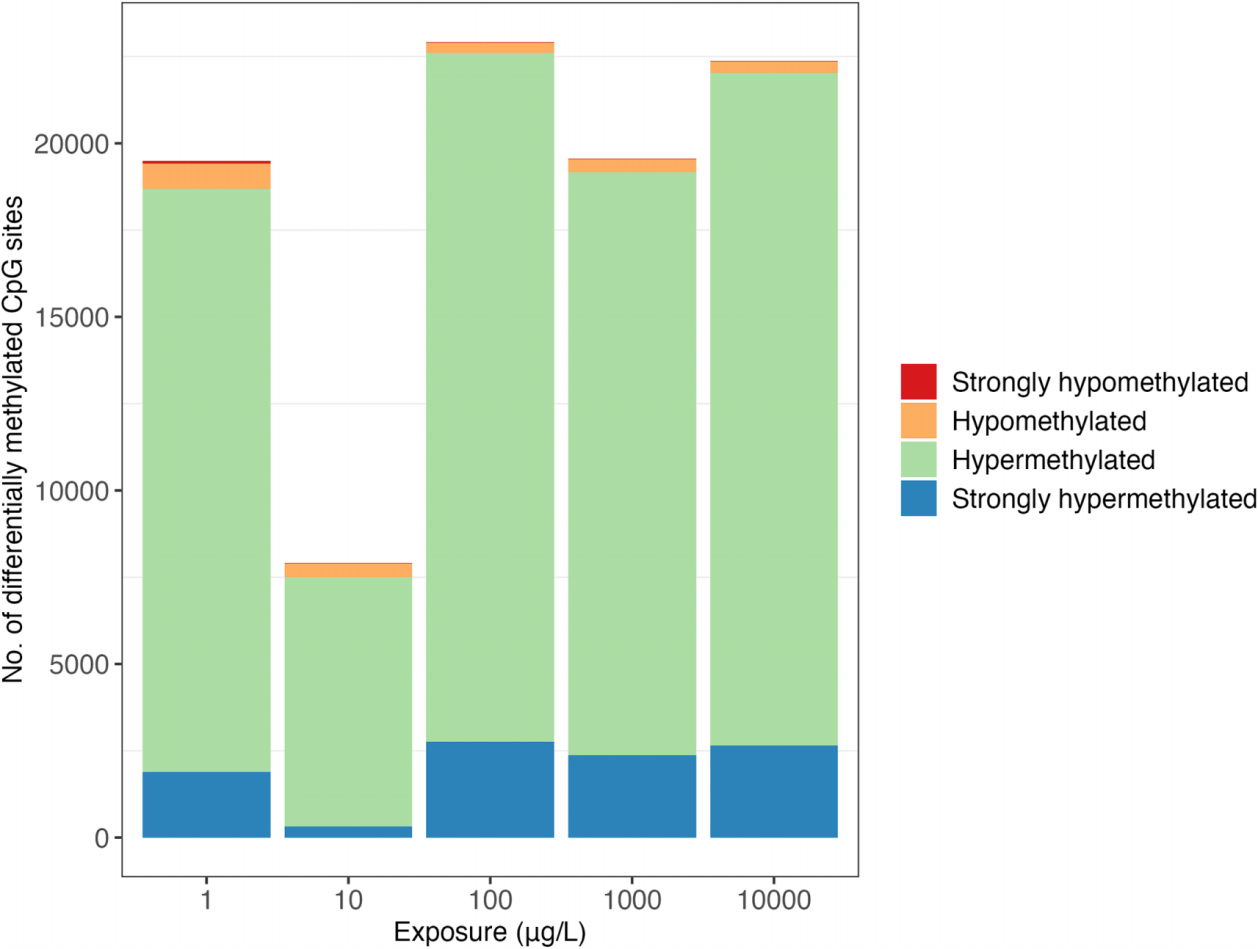
Number of differentially methylated CpG sites (DMCs) in 2-year-old male fish following Pb^2+^ exposure across 1, 10, 100, 1000, and 10 000 μg/l. DMCs with an absolute methylation difference >0.3 were classified as strongly hypermethylated or strongly hypomethylated. DMCs with an absolute difference ≤0.3 were classified as hypermethylated or hypomethylated. Bars represent the total number of DMCs by methylation category (*n* = 3 replicates per exposure group).

A total of 19 489, 7907, 22 923, 19 555, and 22 376 CpG sites were significantly differentially methylated in the brain tissue of the 2-year-old zebrafish following 1, 10, 100, 1000, and 10 000 μg/l Pb^2+^ exposure (Fig. 1). Among them, 100 and 10 000 μg/l resulted in the highest number of differentially methylated CpG sites (DMCs), whereas 10 μg/l had the fewest. More than 85% of the DMCs were hypermethylated across all exposure levels. Hypomethylation accounted for <5% of DMCs. The average genome-wide methylation levels from 0, 1, 10, 100, 1000, and 10 000 μg/l were 78 ± 3%, 83 ± 3% (*P* = .09 compared to control), 82 ± 3% (*P* = .21 compared to control), 85 ± 3% (*P* = .011 compared to control), 84 ± 1% (*P* = .018 compared to control), and 84 ± 1% (*P* = .018 compared to control). These results indicate that exposure to Pb^2+^ levels ≥ 100 μg/l led to significantly increased global methylation compared to the control in aged zebrafish.

**Figure 2:**
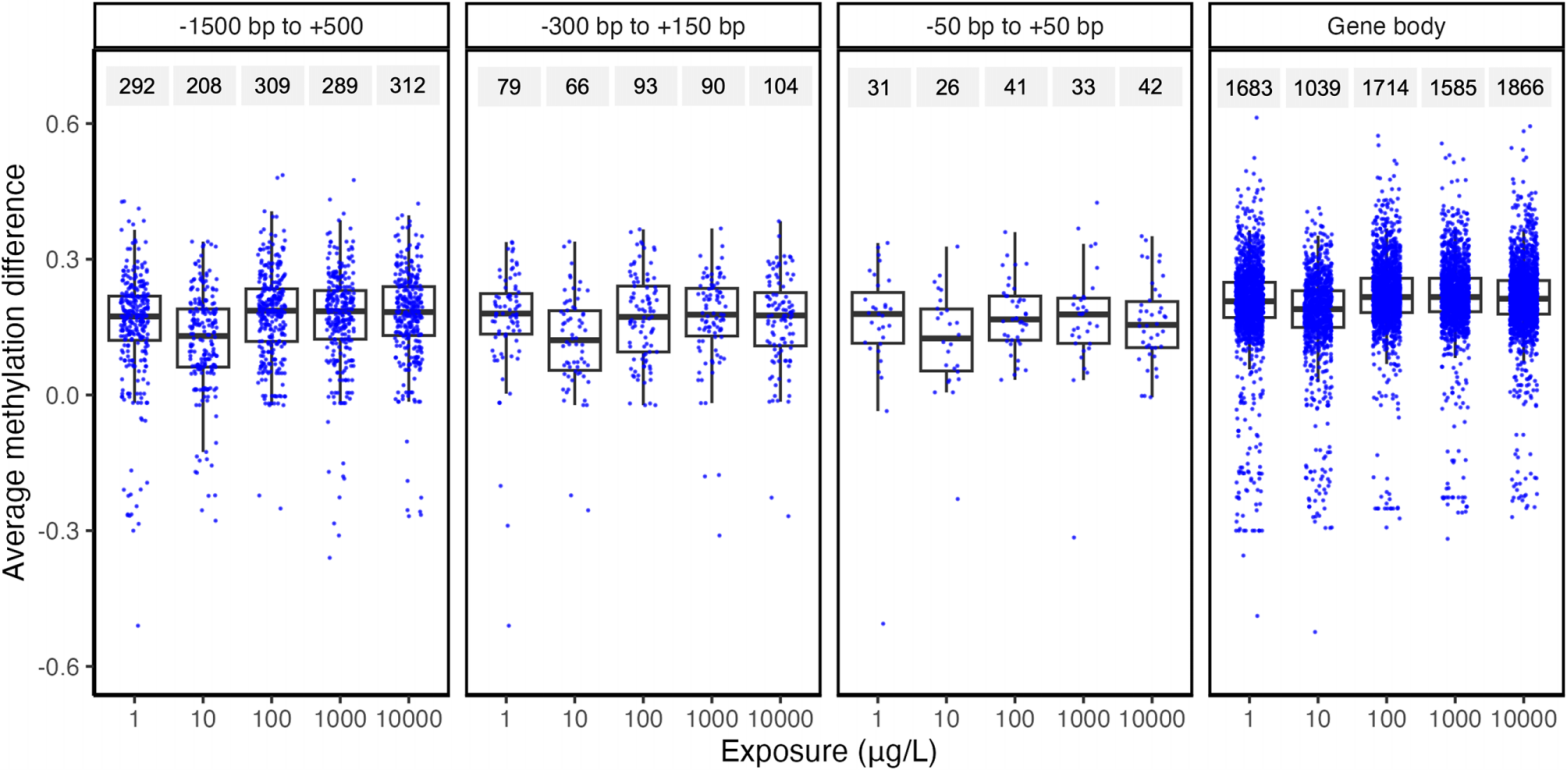
Distribution of average methylation differences per gene across genomic regions (*n* = 3 replicates per exposure group). Genomic locations are defined as −1500 to +500 bp relative to the transcription start site (TSS), −300 to + 150 bp, −50 to +50 bp, and the gene body. Each dot is one DMG, with its value representing the average methylation difference of all differentially methylated CpG sites assigned to that gene within the specified genomic region.

Considering the genomic context of DMCs, we annotated their relative positions within promoter regions and gene bodies. Overall, 85% of DMCs were located in gene bodies. Among promoter-associated regions, 12% were located within −1500 to + 500 bp relative to the transcriptional start site (TSS), including 3% within −300 to +150 bp, and 1% within −50 to +50 bp. Across Pb^2+^ exposure levels of 1, 10, 100, 1000, and 10 000 μg/l, the number of unique differentially methylated genes (DMGs), defined as genes containing at least one DMC, were 1813, 1135, 1853, 1719, and 1992, respectively (Fig. 2). Consistent with global methylation patterns, the 10 and 1 μg/l Pb^2+^ exposures had the lowest methylation difference across all genomic regions compared to the other three exposures.

**Figure 3:**
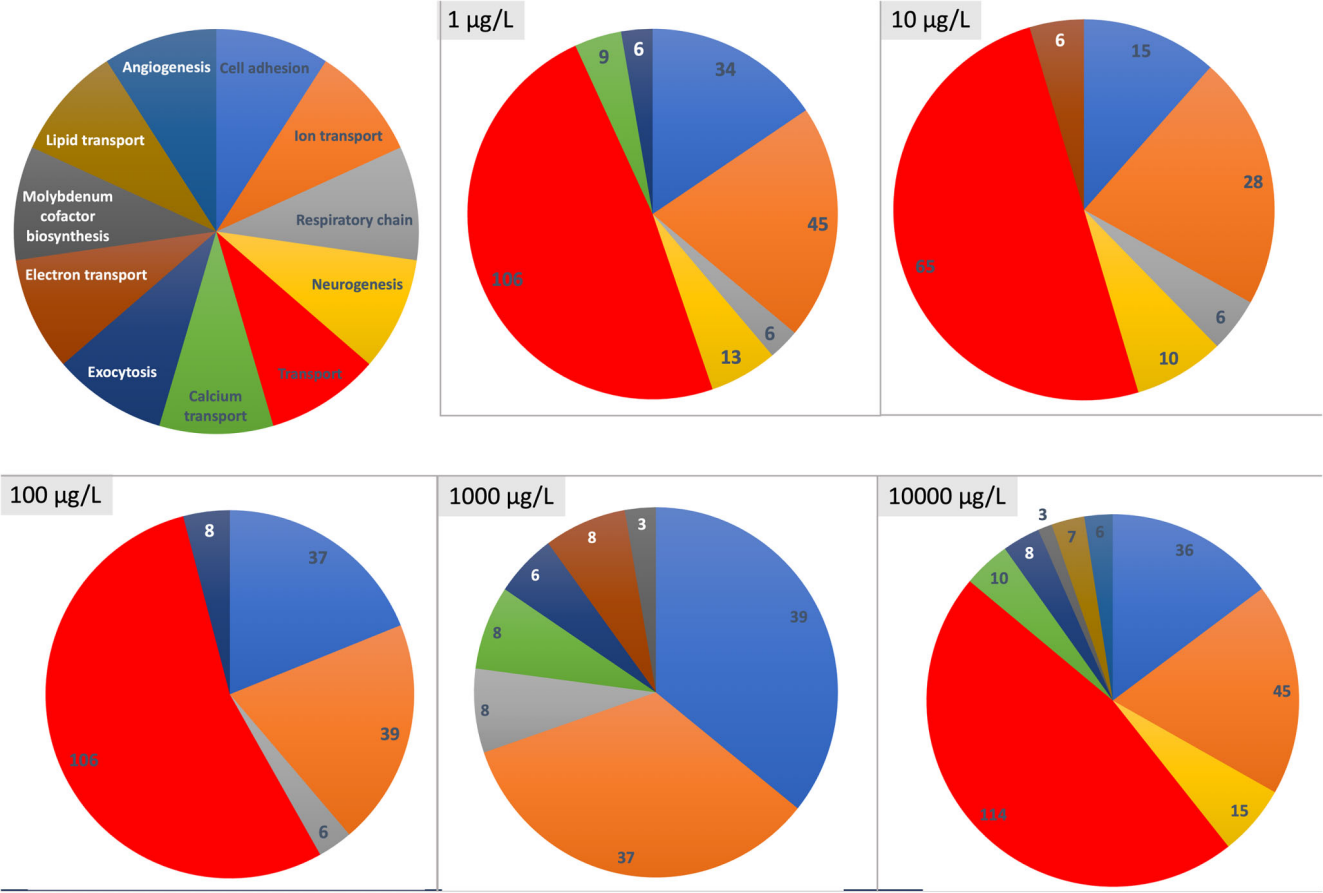
Biological pathways associated with DMGs in 2-year-old zebrafish following 1, 10, 100, 1000, and 10 000 μg/l Pb^2+^ exposure (*n* = 3 replicates per exposure group). The legend for the pie charts is located at the top left of the figure, followed by pie charts representing biological pathways at different Pb^2+^ exposure levels: (A) 1 μg/l, (B) 10 μg/l, (C) 100 μg/l, (D) 1000 μg/l, and (E) 10 000 μg/l.

To identify biological pathways associated with the methylome, DMGs were analyzed using Database for Annotation, Visualization, and Integrated Discovery (DAVID, version 2021) (Fig. 3). Cell adhesion (KW-0130) and ion transport (KW-0406) were the most consistently implicated pathways across all five Pb^2+^ exposure levels. Transport (KW-0813) was another major pathway, observed at all exposure levels except 10μg/l. At the highest Pb level, the methylome was linked to the greatest number of biological pathways, with transport (KW-0813), ion transport (KW-0406), cell adhesion (KW-0130), and neurogenesis (KW-0524) as the four most prominent pathways. Additionally, lipid transport (KW-0445) and angiogenesis (KW-0037) were uniquely present at this exposure level.

**Figure 4:**
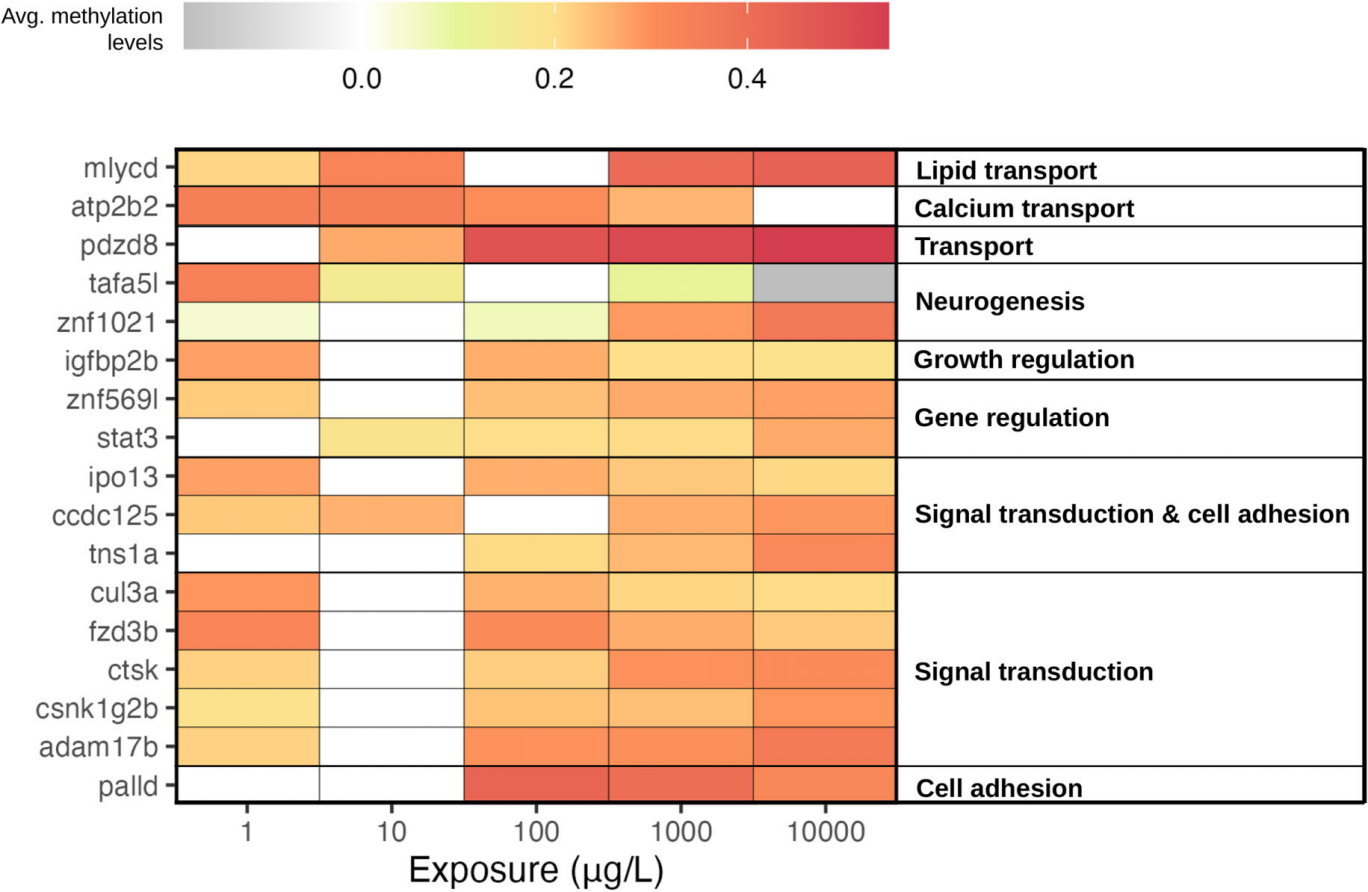
DMGs with average methylation levels correlated with Pb^2+^ exposure (*n* = 3 replicates per exposure group). Genes with an absolute Spearman correlation coefficient of 1, a *P*-value < .05, and an absolute average methylation level ≥ 0.3 at at least one Pb concentration are shown. Color intensity indicates the average methylation level. The left column shows gene symbols, and the associated biological pathways are listed in the right column. Differential methylation for all genes was located within the gene body regions.

To investigate the relationship between methylation changes and Pb^2+^ exposure levels, Spearman correlation analyses were performed between the average DNA methylation levels of individual DMG and the corresponding log-transformed Pb^2+^ concentrations. Genes with an absolute Spearman correlation coefficient of 1, a *P*-value < .05, and an absolute average methylation level ≥0.3 at at least one Pb concentration were included in Figure S1 and Table S1. Among these, genes associated with the biological pathways described above are shown in Fig. 4.

Specifically, genes exhibiting a consistent increase in methylation with increasing Pb^2+^ concentrations included *pdzd8* (PDZ domain containing 8), *stat3* (signal transducer and activator of transcription 3), and tns1a (tensin 1a). Some DMGs showed an overall increasing trend across most Pb^2+^ concentrations, excluding 100 μg/l in *mlycd* (malonyl-CoA decarboxylase) and *ccdc125* (coiled-coil domain containing 125), and 10 μg/l in *znf1021* (zinc finger protein 1021), *znf569l* (zinc finger protein 569, like), *ctsk* (cathepsin K), *csnk1g2b* (gamma 2b casein kinase 1), and *adam17b* (ADAM metallopeptidase domain 17b). In contrast, several genes showed an inverse relationship between Pb^2+^ concentrations and methylation level, including *atp2b2* (ATPase plasma membrane Ca^2+^ transporting 2) and *palld* (palladin, cytoskeletal-associated protein). Other DMGs also exhibited non-monotonic patterns, with *igfbp2b* (insulin-like growth factor binding protein 2b), *ipo13* (importin 13b), *cul3a* (cullin 3a), and *fzd3b* (frizzled class receptor 3b) showing deviations at 10 μg/l, and *tafa5l* (TAFA chemokine-like family member 5, like) at 100 μg/l. All abovementioned genes exhibited differential methylation within gene body regions (Table S1).

**Figure 5:**
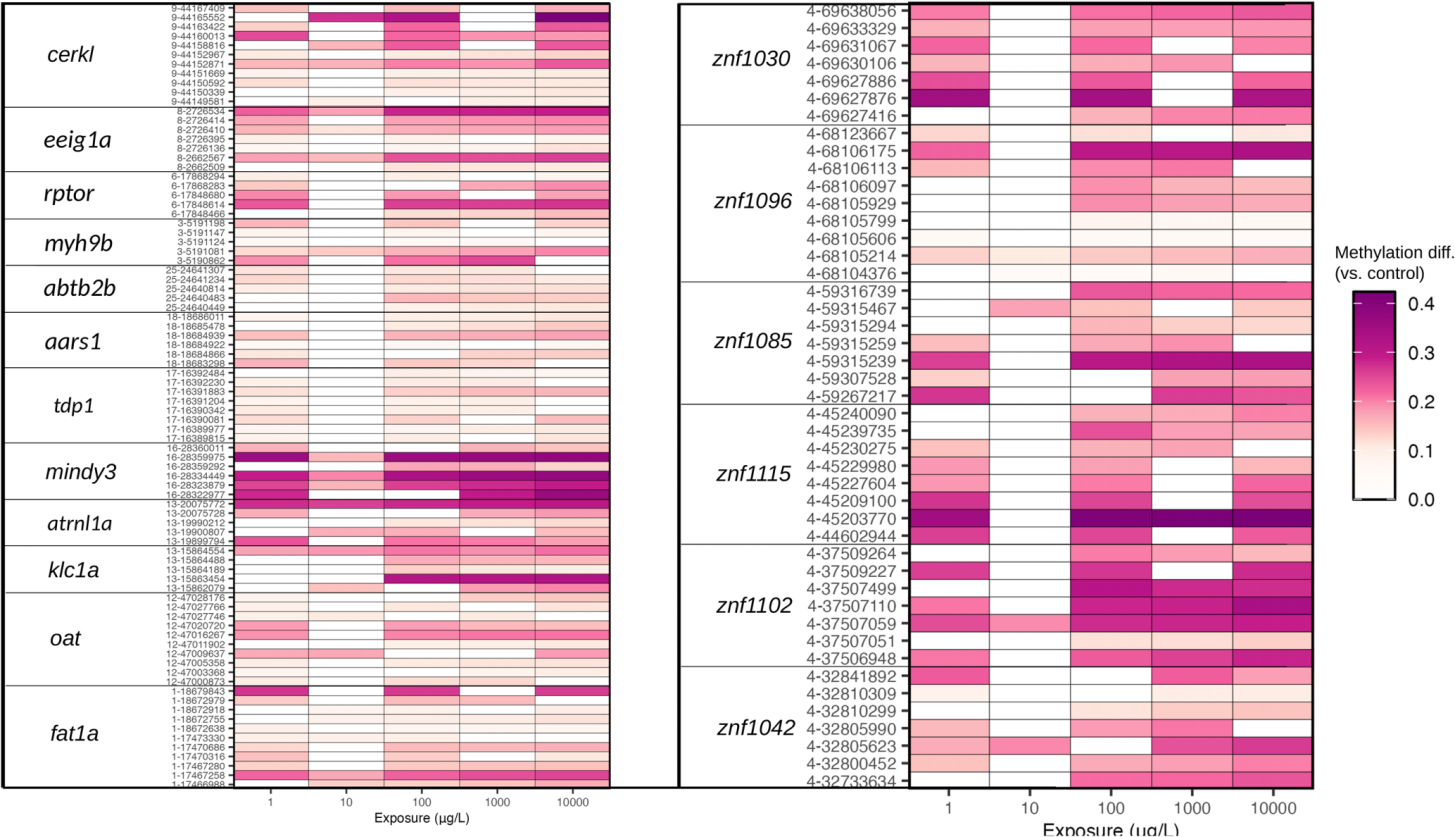
Differentially methylated CpG sites (DMCs) significantly correlated with Pb^2+^ exposure levels and their associated genes (Spearman correlation, *P* < .05) (*n* = 3 replicates per exposure group). CpG sites are denoted by genomic coordinates, where the first number indicates the chromosome and the second number indicates the genomic start position. All DMCs were located within the gene body region. Heatmap colors indicate methylation differences relative to controls across Pb^2+^ exposure concentrations.

We further examined the correlation between methylation levels of individual DMCs and Pb^2+^ exposure concentrations using Spearman correlation analyses. The complete list of correlated DMCs is provided in Table S2. Figure 5 shows eighteen genes containing more than five DMCs that were significantly correlated with more than three Pb^2+^ concentrations (Spearman correlation, *P*-value < .05). These DMCs were all located within gene body regions. Multiple strongly hypermethylated DMCs (methylation difference relative to control >0.3) were located in regions of the genes, including *cerkl* (CERK like autophagy regulator), *mindy3* (MINDY lysine 48 deubiquitinase 3), *klc1a* (kinesin light chain 1a), *atrnl1a* (attractin-like 1a), *eeig1a* (estrogen-induced osteoclastogenesis regulator 1a), *tbc1d1* (tyrosyl-DNA phosphodiesterase 1), *rptor* (regulatory associated protein of MTOR, complex 1), *myh9b* (myosin heavy chain 9b), *fat1a* (FAT atypical cadherin 1a), and several zinc finger (*znf*) genes, including *znf1115, znf1030, znf1102, znf1085*, and *znf1096*. In contrast, genes such as *aars1* (alanyl-tRNA synthetase 1), *oat* (ornithine aminotransferase), *abtb2b* (ankyrin repeat and BTB (POZ) domain containing 2b), *tdp1* (tyrosyl-DNA phosphodiesterase 1), and *znf1042* contained DMCs with methylation differences between 0.09 and 0.29. Most DMCs showed the lowest degree of change at 10 μg/l exposure.

The identified DMGs showed limited overlap with previously reported transcriptomic responses [15] (Table S3). Table 1 summarizes 20 genes that exhibited both differential methylation and differential expression at more than one Pb^2+^ concentration across 100 to 10 000 μg/l, ranked from the highest to the lowest magnitude of methylation changes. Among them, seven genes showed a pattern of hypermethylation accompanied by downregulation, including *rab11bb* (RAB11B, member RAS oncogene family), *vps39* (VPS39 subunit of HOPS complex), *ppfia4* (PTPRF interacting protein alpha 4), *pip5k1c* (Phosphatidylinositol-4-phosphate 5-kinase, type I, gamma b), *lrp8* (LDL receptor-related protein 8), *aopep* (Aminopeptidase O), and *wbp11* (WW domain binding protein 11). One gene, *ctsa* (Cathepsin A), exhibited hypomethylation with upregulation. The remaining genes displayed both hypermethylation and upregulation. Notably, *fndc3a* (Fibronectin type III domain containing 3A), *shank1* (SH3 and multiple ankyrin repeat domains 1), and *dock3* (Dedicator of cytokinesis 3) showed the largest methylation changes (up to 0.38), accompanied by strong upregulation (log₂FC ≥ 1.5). Most of these genes did not follow a sequential dose–response trend between methylation levels and Pb^2+^ concentrations. Exceptions include *stat3*, which showed a consistent increase in average methylation, and *msantd4*, which showed slight CpG site-specific correlation across non-consecutive Pb^2+^ levels. Among these genes, only *ptprsa* (PTPRF interacting protein alpha 4), *msantd4* (Myb/SANT-like DNA-binding domain containing 4 with coiled-coils), and *wbp11* contained DMCs located in promoter-associated regions, whereas the remaining overlapped genes exhibited methylation changes within gene body regions.

**Table 1:** Genes with overlapping differential methylation and expression at the same Pb^2+^ concentration, corresponding to peak expression level.

Gene name	Full gene name	Genomic location	Differential methylation changes (Pb^2+^)	Log2 fold changes (Pb^2+^)
*fndc3a*	Fibronectin type III domain containing 3A	Gene body	0.38 (10 000)	1.5 (10 000)
*shank1*	SH3 and multiple ankyrin repeat domains 1	Gene body	0.34 (10 000)	1.5 (10 000)
*dock3*	Dedicator of cytokinesis 3	Gene body	0.32 (10 000)	1.3 (10 000)
*rab11bb*	RAB11B, member RAS oncogene family	Gene body	0.27 (10 000)	–1.0 (10 000)
*plxna2*	Plexin A2	Gene body	0.26 (100)	1.06 (100)
*frs3*	Fibroblast growth factor receptor substrate 3	Gene body	0.25 (10 000)	1.6 (10 000)
*ptprea*	protein tyrosine phosphatase receptor type Ea	Gene body	0.24 (10 000)	1.2 (10 000)
*myh9b*	Myosin, heavy chain 9b, non-muscle	Gene body	0.23 (10 000)	1.3 (10 000)
*vps39*	VPS39 subunit of HOPS complex	Gene body	0.23 (10 000)	–1.1 (10 000)
*stat3*	Signal transducer and activator of transcription 3	Gene body	0.21 (1000)	1.58 (1000)
*ppfia4*	PTPRF interacting protein alpha 4	Gene body	0.21 (1000)	–1.1 (1000)
*ptprsa*	Protein tyrosine phosphatase receptor type Sa	−1500 to +500 bp to TSS; gene body	0.19 (100)	1.5 (100)
*pip5k1c*	Phosphatidylinositol-4-phosphate 5-kinase, type I, gamma b	Gene body	0.19 (1000)	–1.4 (1000)
*msantd4*	Myb/SANT-like DNA-binding domain containing 4 with coiled-coils	−1500 to +500 bp to TSS	0.18 (10 000)	2.7 (10 000)
*adam11*	ADAM metallopeptidase domain 11	Gene body	0.18 (1000)	2.5 (1000)
*rab21*	RAB21, member RAS oncogene family	Gene body	0.18 (10 000)	2.4 (10 000)
*lrp8*	LDL receptor related protein 8	Gene body	0.17 (100)	–0.8 (100)
*aopep*	Aminopeptidase O (putative)	Gene body	0.16 (100)	–1.3 (100)
*wbp11*	WW domain binding protein 11	−1500 to +500 bp to TSS	0.15 (100)	–1.7 (100)
*ctsa*	Cathepsin A	Gene body	–0.12 (1000)	1.5 (1000)

## Discussion

Our findings reveal that Pb^2+^ exposure at concentrations ≥ 100 μg/l induces widespread gene body methylation in the brains of aged zebrafish. Gene body methylation is an epigenetic feature that has been proposed to play roles in silencing repetitive DNA elements, regulating transcriptional elongation, modulating alternative splicing, and influencing other regulatory elements such as enhancers or insulators [20]. In contrast to promoter methylation, where strong methylation is typically associated with transcriptional repression, gene body methylation has been reported to correlate with active transcription in moderately to highly expressed genes [21]. Additionally, it has been found that methylation at specific CpG sites within gene bodies is strongly associated with aging in zebrafish tissues, including the caudal fin [22]. These findings highlight the importance of Pb-associated methylation changes at specific genomic sites, which may have functional consequences relevant to aging-related neurological disorders.

We observed limited overlap between genes showing both differential expression and differential methylation. Similarly, previous analyses of adult zebrafish brain methylomes in comparison with gene differential expression profiles show limited overlap [23]. Notably, in the adult zebrafish brain methylome dataset, the DMCs within the genes that were both differentially expressed and methylated were also predominantly located in gene bodies or intergenic regions, rather than in promoters [23]. This pattern aligns with previous findings that the functional role of gene body methylation may depend on cell differentiation status [12, 24]. Given that our data were derived from whole-brain homogenates, potential cell-type or region-specific methylation patterns may have been masked. In addition, the nominal exposure concentrations may not reflect the effective uptake of Pb^2+^ in tissues, making the Pb-induced responses not apparent in dose–response pattern. Nevertheless, the genes that exhibited apparent methylation trends with Pb^2+^ exposure in our study may represent robust and integrative signals under aged conditions. We therefore focused on these genes in the following discussion.

Since our previous study reported locomotor and anxiety-like behavioral changes in aged zebrafish following non-monotonic Pb^2+^ exposure [15], we examined DMGs that may be associated with these behavioral phenotypes. These genes included *tdp1, klc1, tns1a, shank1, ptprsa, plxna2, aopep*, and *adam11*. Interestingly, the last five DMGs were also differentially expressed, with *tns1a* and *shank1* showing the highest differential expression at 10 000 μg/l Pb^2+^, *ptprsa, plxna2*, and *aopep* at 100 μg/l Pb^2+^, and *adam11* at 1000 μg/l Pb^2+^. *Tdp1*, which has been implicated in adult locomotor activity, has been shown to cause mild locomotor impairment when deleted [25]. Consistent with our earlier findings implicating cytoskeletal organization [15], *klc1* [26]*, tns1a* [27], and *shank1* [28] are involved in the tubulin-associated cytoskeletal network specifically at 10 000 μg/l Pb^2+^. *Klc1* encodes a subunit of kinesin-1 motor complex responsible for anterograde axonal transport, a process critical for proper neurotransmission. S*hank1*, a gene primarily expressed in the synapse, was hypermethylated only at 10 000 μg/l and upregulated at both 100 and 10 000 μg/l Pb^2+^. Hypermethylation of *shank1, shank2*, and *shank3* has been observed in brain and blood samples of epilepsy patients [29]. Among the remaining DMGs, *ptprs* deletion leads to early-life neurological deficits, motor dysfunction, impaired synaptic input, and high postnatal mortality [30], as well as memory impairment during juvenile stage [31]. *Plxna2* encodes Plexin A2, a receptor involved in plexin signaling pathways that regulate neuronal development, including proliferation, migration, and positioning [32]. Both animal and human clinical observations have linked *plxna2* to motor function and cognitive ability [33, 34]. In addition, single-nucleotide polymorphisms and variant studies have associated *plxna2* with cognitive-related disorders, including autism spectrum disorder [35], schizophrenia [36], and cognitive aging [37]. *Aopep* encodes a zinc-dependent aminopeptidase involved in synaptogenesis and neural maintenance [38]. Variants in *aopep* have been identified in individuals with autosomal recessive dystonia [39], suggesting its relevance to motor dysfunction. Lastly, *adam11* encodes a membrane-anchored protein implicated in neurogenesis, learning, and motor coordination [40].

Among the genes showing clear methylation trends across Pb^2+^ concentrations, several were involved in pathways critical to neuronal signal transduction and ion transportation, most notably calcium homeostasis and Wnt signaling. Several DMGs involved in calcium signaling showed dose–response patterns with Pb^2+^ exposure, including *fat1a, atp2b2*, and *pdzd8. Fat1a* showed non-monotonic methylation changes at CpG sites across increasing Pb^2+^ concentrations, whereas average *pdzd8* methylation was directly correlated and *atp2b2* methylation was inversely correlated with Pb^2+^ exposure levels. Among them, *atp2b2* encodes a plasma membrane ATPase that regulates intracellular Ca^2+^ homeostasis in the brain [41]. Altered methylation and expression of *atp2b2* have also been identified in the prefrontal cortex of patients with schizophrenia [42]. *Pdzd8* has previously exhibited hypomethylation in blood and brain samples from Alzheimer’s disease patients of both sexes [43]. In contrast, our study found increasing hypermethylation of *pdzd8* with higher Pb^2+^ concentrations in the aged male zebrafish brain. While most studies on Pb-induced disruption of calcium homeostasis focus on physiological ion competition, other findings support the possibility of epigenetic regulation as an additional mechanism. These findings suggest that Pb^2+^ exposure may disrupt calcium homeostasis in the aged brain through epigenetic modifications of key calcium signaling genes.

Beyond calcium signaling, differential methylation of *fzd3b, csnk1g2b*, and *adam17b* all point to Wnt signaling as another pathway potentially disrupted by Pb^2+^ exposure. Among them, *fzd3b* exhibited hypomethylation, whereas *csnk1g2b* and *adam17b* showed hypermethylation with increasing Pb^2+^ concentrations. *Fzd3d* encodes a Frizzled receptor that mediates both canonical and non-canonical Wnt signaling. In the canonical pathway, CSNK1G2 activates the Wnt signaling by phosphorylating LRP co-receptors, such as LRP5 and LRP6. In our study, we also identified a member of the LRP family, *lrp8*, as a DMG. *Lrp8* was hypermethylated and downregulated at Pb^2+^ concentrations ≥ 100 μg/l. Although LRP8 is less well-characterized than LRP5/6 in Wnt signaling, its structural similarity suggests potential involvement. Additionally, *adam17b* gene expression has been activated by canonical Wnt signaling through chromatin remodeling and histone demethylation in colorectal cancer cells [44]. On the other hand, the locomotor-related gene *shank1* is involved in synapse maturation by modulating noncanonical Wnt signaling during neuronal development [45]. In line with our finding, a previous study of brain samples from Parkinson’s disease patients reported that genes involved in Wnt signaling showed abnormal DNA methylation and expression, although the study did not specify whether the canonical or non-canonical Wnt pathways were predominantly affected [46]. While much of the existing research has focused on how developmental Pb^2+^ exposure inhibits Wnt signaling and leads to premature neural differentiation [47] and altered synaptogenesis [48], our study is the first to identify Wnt-related genes in aged organisms whose methylation and/or expression may be altered in response to Pb^2+^ exposure.

In addition to Wnt signaling, several DMGs showing consistent methylation trends with increasing Pb^2+^ exposure—*adam17b, klc1*, and *ptprs*—have been implicated in Alzheimer’s disease through amyloid- or tau-regulated pathways. *Adam17b* encodes a disintegrin and metalloprotease that cleaves the amyloid precursor protein (APP) at the α-secretase site. By promoting non-amyloidogenic APP processing and reducing amyloid-β accumulation, ADAM17 has been proposed as a therapeutic target for Alzheimer’s disease [49]. In our analysis, *adam17b* displayed a consistent increase in methylation with Pb^2+^ concentrations ≥ 100 μg/l, although it was not differentially expressed at the transcript level [15]. While studies have demonstrated that *adam17b* expression can be suppressed by miRNA in glioma cells [50], the specific role of DNA methylation in regulating *adam17b* expression in neural-related tissues or cells remains unclear. Another DMG associated with amyloid-β accumulation is *klc1*. Reduced expression of KLC1 protein has been shown to impair APP trafficking, thereby contributing to amyloid plaque formation in Alzheimer’s disease [51] [52]. In contrast to *adam17* and *klc1a*, which influence amyloidogenic processes, *ptprs* has been more directly linked to tau pathology. *Ptprs* encodes a member of the protein tyrosine phosphates (PTP) family, whose phosphatase activity can prevent tau hyperphosphorylation [53], suggesting a neuroprotective role in early-stage tau pathology in Alzheimer’s disease. In our study, *ptprs* exhibited both hypermethylation and upregulation, though the pattern was not clearly Pb^2+^ dose-dependent. The altered methylation patterns observed in, *klc1a*, and *ptprs* suggest that key Alzheimer’s disease–related genes may serve as epigenetically modifiable biomarkers of Pb²⁺ exposure, with potential implications for movement and broader neurological dysfunctions.

Among the DMGs showing overlapping differential expression, *msantd4* and *wbp11* were further examined due to their specific CpG hypermethylation within the promoter-proximal region, −1500 bp to + 50 bp relative to the TSS. *Msantd4* encodes a DNA-binding protein expressed during zebrafish embryonic development and in adult tissues, predominantly in the male brain, as well as in the human brain during fetal development [54]. Knockdown of *msantd4* has been shown to result in nervous system malfunction and impaired brain ventricle formation [54]. *Wbp11* encodes a nuclear protein involved in RNA splicing, mRNA processing, and rRNA processing [55]. Although both genes are relatively understudied, their promoter-proximal methylation changes and differential expression in response to Pb^2+^ exposure suggest they may be involved in epigenetic regulation in the aged brain.

This study employed a controlled experimental design to examine Pb^2+^ exposure and its epigenetic impacts in an aged zebrafish model. Pb^2+^ exposure in aged zebrafish predominantly induces gene body-localized DNA methylation with minimal overlap with differentially expressed genes. Several of these DMGs are associated with motor functions, including *shank1* in response to high Pb^2+^ insult, and *ptprsa, plxna2*, and *aopep* at 100 μg/l Pb^2+^, consistent with previously observed Pb-induced behavioral alterations. Given that Pb^2+^ exposure at concentrations ≥ 100 μg/l increased global methylation in the aged brain, its potential contribution to locomotor dysfunction and neurodegeneration-related diseases through gene body methylation-mediated regulatory mechanisms warrants further investigation. Future studies incorporating long-term Pb^2+^ exposure and direct quantification of Pb uptake will be critical for clarifying gene-specific dose–response relationships and their functional consequences.

## Materials and methods

### Animals

Zebrafish (AB wildtype fish line) were housed in a recirculating aquatic housing system. Routine husbandry and care were provided by trained laboratory personnel. All animal procedures were approved by the Animal Care and Use Committee at Wayne State University (protocol number 19-02-0938) and were conducted in accordance with the NIH *Guide for the Care and Use of Laboratory Animals*. Zebrafish husbandry followed established husbandry protocol and guidelines described by Westerfield [56].

### Acute Pb^2+^ exposure

Two-year-old zebrafish were exposed to waterborne lead acetate (Sigma-Aldrich, St. Louis, MO, USA) at nominal concentrations of 1, 10, 100, 1000, and 10 000 μg/l, or to a fish water control for five consecutive days. The exposure levels spanned environmentally relevant concentrations (1–100 µg/l) [57, 58] through higher levels up to 10 000 µg/l to assess acute toxicity responses to high-insult conditions. Four aged zebrafish were assigned to each exposure group. Each exposure group was conducted in 1.5 l beakers containing 800 ml exposure solution. To maintain water quality, 80% of the water was renewed daily with freshly prepared exposure solutions. As previous studies have reported sex-dependent behavioral impairment and transcriptomic alterations following Pb^2+^ exposure [16, 17], only aged male zebrafish were used in this study.

### Genomic DNA isolation and library preparation

Three adult male zebrafish from each exposure group were selected for WGBS analysis. Fish were euthanized using 16.7 mg/ml tricaine methanesulfonate solution. Brains were dissected and preserved in RNALater^TM^ (Thermo Fisher, Waltham, MA, USA) at −80°C until nucleic acid extraction. Prior to extraction, RNALater^TM^ was removed from the tissue. Genomic DNA extraction was extracted using the Quick-DNA/RNA^TM^ Miniprep plus Kit (Zymo, Irvine, CA, USA) according to the manufacturer’s protocol. Briefly, each individual brain sample was pretreated with 95 μl DNase/RNase-free water, 95 μl PK digestion buffer, and 10 μl proteinase K, followed by incubation at 55°C for 30 min and an additional incubation at 94°C for 20 min. After incubation, samples were centrifuged at 10 000 × *g* for 30 s. The resulting supernatant was mixed with DNA/RNA lysis buffer at a 1:1 ratio and loaded onto spin columns for DNA purification. DNA concentrations were quantified using the Qubit dsDNA High Sensitivity Assay Kit (Thermo Fisher, Waltham, MA, USA). DNA methylation library preparation was performed by the University of Florida Interdisciplinary Center for Biotechnology Research core using Illumina NEB Enzymatic-based Methylation library preparation kits (Ipswich, MA, USA) according to the manufacturer’s instructions.

### WGBS sequencing and bioinformatics

Paired-end sequencing of the WGBS libraries was performed on a NovaSeq 6000 platform (S4 flow cell, 2 × 150 bp, one lane) (Illumina, CA, USA) at the University of Florida Interdisciplinary Center for Biotechnology Research core. Triplicates for each exposure group were sequenced, and each biological sample contained ~120 million paired-end reads. Short reads were trimmed using Trimmomatic (v 0.36) [59], and quality control was assessed on both original and trimmed reads using FastQC (v 0.11.4) [60] and MultiQC [61]. Trimmed reads were aligned to the zebrafish genome (danRer, GRCz11 assembly) using BSMAP [62]. Methylation calling was performed with cscall v1.0 [63] using the danRer.GRCz11-CG index.

A CpG site was included in downstream analysis if it reached a minimum coverage of 20 reads in at least two samples. Differential methylation analysis was conducted using the “mcomp” program and part of moabs package [64]. Differentially methylated CpG sites (DMCs) were defined as CpG with a *P*-value < .01 when comparing methylation rates between control and exposed groups. DMCs showing a methylation difference above 0.3 were classified as strongly hypermethylated or strongly hypomethylated, with those with difference ≤0.3 were classified as hypermethylated or hypomethylated. The average genome-wide methylation was calculated as the mean of all CpG methylation rates across the genome and compared between groups using a two-sample *t*-test.

DMGs were called when at least one DMC was located within the gene body or within promoter regions defined as −1500 to +500 bp, −300 to +150 bp, or −50 to +50 bp relative to the TSS [65]. The average difference in methylation level across all DMCs within each gene was then calculated between control and exposed groups. Genes were classified as DMGs if the *P*-value < .01 when comparing between control and exposed groups using a two-sample *t*-test. DMGs were categorized as strongly hypermethylated or strongly hypomethylated if the absolute average difference exceeded 0.3, and as hypermethylated or hypomethylated if ≤0.3.

### Pathway analysis

DMGs were subjected to gene enrichment and functional annotation clustering analysis using the Database for Annotation, Visualization, and Integrated Discovery (DAVID, version 2021) [66, 67]. UniProtKB Keywords (UP_KW) were considered significantly enriched when the Benjamini-adjusted false discovery rate was <0.05.

## Supplementary Material

dvag023_Supplemental_Files

## Data Availability

622 Data is included in the Supplemental files and will be made available upon reasonable request to the corresponding author, Dr. Tracie Baker, tracie.baker@ufl.edu.
