# Supplementary material for: Gene-specific DNA methylation responses to Pb2+ exposure in aged male zebrafish and associations with locomotor dysfunction
Source: Environ Epigenet. 2026 Jun 23;12(1):dvag023. doi: 10.1093/eep/dvag023 (PMC13411783; doi:10.1093/eep/dvag023)
Supplement: dvag023_Supplemental_Files [file dvag023_supplemental_files.zip › 130726080757_Supplementary_figure.docx]

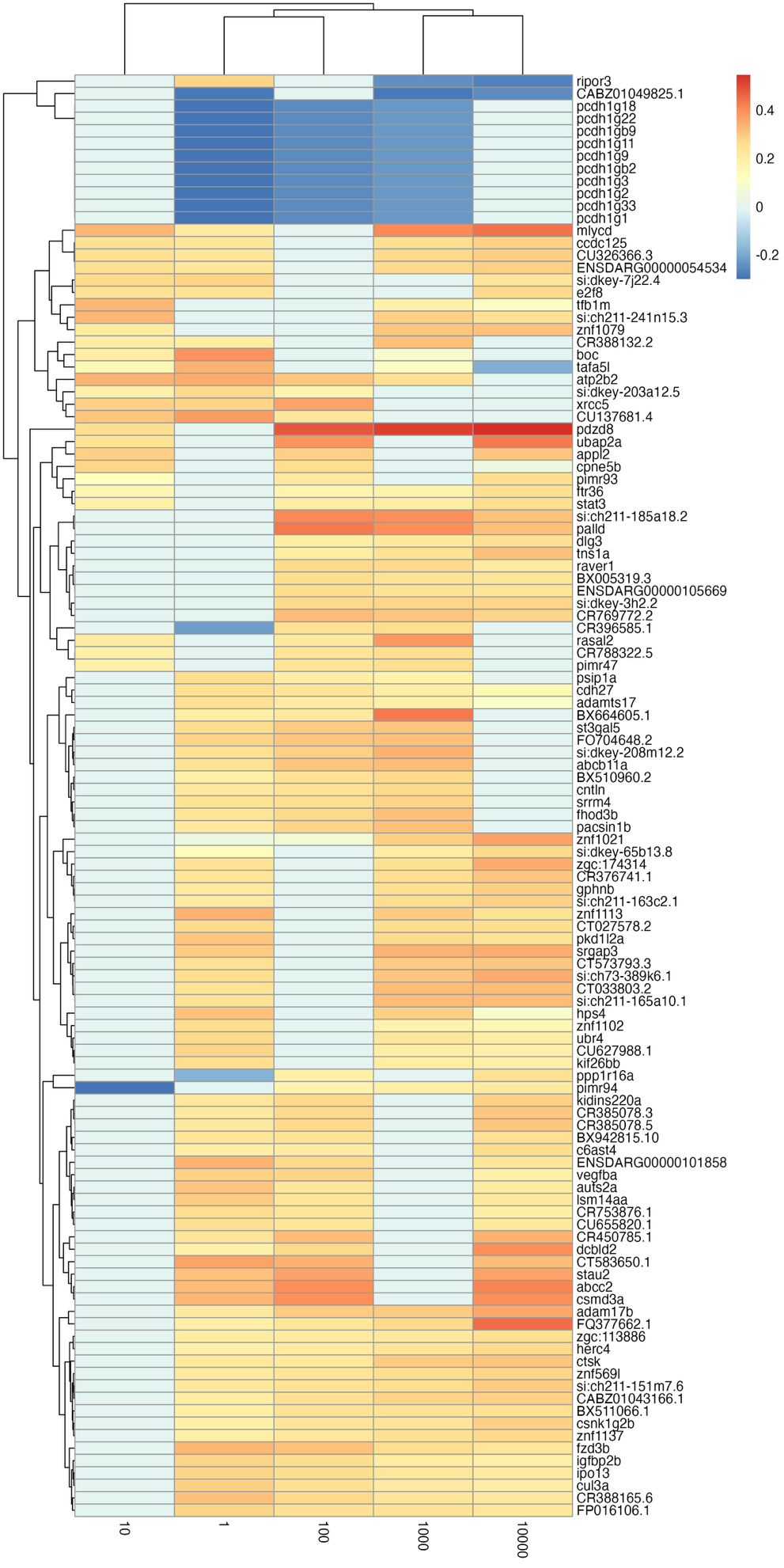


Figure S1. Heatmap clustering of differentially methylated genes (DMGs) whose average methylation levels were correlated with Pb²⁺ exposure. Genes with an absolute Spearman correlation coefficient = 1, a p-value < 0.05, and an absolute average methylation level ≥ 0.3 at at least one Pb concentration are shown. **Color intensity indicates the average DNA methylation level. The right column shows gene symbols, and the numbers at the bottom indicate Pb exposure concentrations (**µg/L).
